# Molecular and Synaptic Signatures in Mouse Models of Late‐Onset Alzheimer’s Disease Independent of Amyloid and Tau Pathology

**DOI:** 10.1002/alz.086529

**Published:** 2025-01-03

**Authors:** Adrian L Oblak

**Affiliations:** ^1^ Stark Neurosciences Research Institute, Indiana University School of Medicine, Indianapolis, IN USA

## Abstract

**Background:**

MODEL‐AD (Model Organism Development and Evaluation for Late‐onset AD) is developing, characterizing, and distributing novel mouse models expressing humanized, clinically relevant genetic risk factors. Models expressing human‐relevant risk genetic risk factors are expected to better phenocopy LOAD than widely used transgenic models.

**Method:**

Here, two genetic risk factors APOE4 and Trem2*R47H, were incorporated into C57BL/6J (B6) mice along with humanized amyloid‐beta to produce the LOAD2 model. LOAD2 and control mice were aged up to 24 months with some being provided in the absence or presence of normal chow or a high fat/high sugar diet (LOAD2 HFD) from two months of age. A phenotyping pipeline was employed to evaluate disease outcomes observed in human patients, including in vivo imaging, brain and blood biomarker and cytokine analyses, multi‐omics (transcriptomics and proteomics), neuropathology and behavior.

**Result:**

By 18 months, unlike control mice (e.g., LOAD2 mice fed a control diet, CD), LOAD2 HFD mice presented subtle but significant loss of neurons in the cortex, elevated levels of insoluble AΒ42 in the brain, and increased plasma neurofilament light chain (NfL). Transcriptomics and proteomics showed changes in gene/proteins relating to a variety of disease‐relevant processes including lipid metabolism and synaptic function. In vivo imaging revealed an age‐dependent reduction in brain region volume (MRI) and neurovascular uncoupling (PET/CT). LOAD2 HFD mice also showed a learning deficit based on a Touchscreen cognitive assay.

**Conclusion:**

Despite the absence of hallmark amyloid and Tau pathologies, collectively these data support the use of LOAD2 HFD mice reveal this model as important for preclinical studies that target other features of LOAD independent of amyloid and tau.

